# Long-metallic-strip array with parasitic rings: an efficient metasurface for dual-broadband electromagnetic window at large angles

**DOI:** 10.1515/nanoph-2025-0157

**Published:** 2025-10-16

**Authors:** Tiefu Li, Jinwei Chen, Yuxiang Jia, Xinmin Fu, Yajuan Han, Jie Yang, Zhaotang Liu, Chang Ding, Cunqian Feng, Jiafu Wang

**Affiliations:** Air and Missile Defense College, Air Force Engineering University, Xi’an 710051, China; 669271 Aerospace Metamaterials Laboratory of SuZhou Laboratory, Suzhou 215004, China; Shaanxi Key Laboratory of Artificially-Structured Functional Materials and Devices, Air Force Engineering University, Xi’an 710051, China

**Keywords:** metasurface, dual-band, electromagnetic window, large angles, plasma-like oscillation

## Abstract

The long-metallic-strip (LMS) array proposed by J. B. Pendry has been widely studied in electromagnetic windows. However, it could only work for the single-band window, not the dual-band one, due to the plasma-like oscillation attenuating with increasing frequency. To solve the problem, we have analyzed the condition of EM windows and then proposed to introduce “parasitic” rings (PRs) into the LMS array. The design is called the LPR metasurface, which could open the dual-broadband window at large incident angles (60°–85°). In C band, the LPR metasurface could conserve the LMSs’ plasma-like oscillation, thus opening a broadband window for transverse-electric polarization. In K band, where the plasma-like oscillation has almost disappeared, the PR generates a capacitive resonance. It could open an additional window at large angles and provide a great out-of-band suppression concurrently. Unlike related studies, the PRs here are structurally easy and integrated into the LMSs. It has conserved the LMSs’ simplicity and continuity, thus could better meet processing and protective materials. Additionally, with the great performance at large angles, the LPR metasurface may find wide applications in hypersonic aircraft radars, 5G/6G base stations, and others.

## 1 Introduction

Electromagnetic (EM) window is the general name of radomes. As the important structure-function integrated equipment in active radio frequency (RF) systems, it has been widely used in phased array radar, 5G/6G base stations, and others. With traditional anti-reflection (AR) techniques (AR films and gradient AR layers) poorly meeting applications, metasurfaces have been explored well in EM windows [1], [2], [3], [4]. Among them, with the plasma-like oscillation and simple structure, the long-metallic-strip (LMS) array proposed by J. B. Pendry in 1996 has been the most widely studied, especially for transverse-electric (TE) polarization [5], [6], [7], [8], [9], [10], [11], [12], [13], [14], [15], [16], [17], [18].

Because the plasma-like oscillation has attenuated with increasing frequency, the LMS array could only work for the single-band window, not the dual-band one [6], [7], [8], [9], [10], [11], [12], [13], [14], [15], [16]. However, the dual-band window has been greatly required in some special scenarios, such as multi-mode guidance and shared-aperture wireless communications. Therefore, lots of researchers have introduced EM resonators into the array to open an additional window. For example, Da Li et al. have combined the metallic slot and lumped capacitor with the LMS array, thus designing a dual-band bandpass frequency selective surface (FSS). The FSS could open two transparent windows at around 1.0 GHz and 15.0 GHz, respectively. In 2023, Qiang Cheng et al. introduced metallic grid and oblique dipole antennas into the LMSs to propose a dual-band transparent metasurface [17]. With the glasses as the substrate, the design is not only optically transparent, but also has two EM windows in 2.2–3.2 GHz and 4.4–5.2 GHz, respectively [18].

Although introducing additional resonators into the LMSs has shown great superiority in dual-band windows, there are some problems. First, the additional resonators are generally complex structures. It has destroyed the LMS array’s simplicity, thus increasing the processing difficulty. The problem would be particularly serious in HF applications, such as those at the visible-light and terahertz frequencies. Moreover, the resonators and LMSs are not even on the same surface in some designs, like Reference [17], which would worsen it. Second, the introduced resonators are completely structurally independent of the LMSs, which has destroyed the LMSs’ continuity [19], [20], [21], [22], [23]. For a wider transparent bandwidth, the metasurface should be loaded inside the substrate sometimes (generally in the middle layer) [10], [13], [14], [15]. However, to protect the internal structure, EM windows are always composed of materials with protective properties. Such as ceramic matrix composites (CMCs), which have the high mechanical strength, temperature resistance, and others [24], [25], [26]. For these protective materials, the metasurface could only be loaded inside them through some special techniques, like the textile metasurface, which has required the metasurface units to be continuous [19], [20], [21], [22], [23]. Third, most designs work poorly, or even cannot work at large incident angles (≥60°). For example, with the maximum effective angle of only 45°, Reference [17] couldn’t work at large angles. Meanwhile, the effective large-angle bandwidth of Reference [18] is also very limited. It’s mainly because of the impedance mismatch between the dielectric and air, which has deteriorated with increasing angles. Compared with transverse-magnetic (TM) polarization, the problem would be much more serious at TE one, due to the lack of Brewster Angle [27], [28]. However, EM waves would be generally incident at large angles in some special applications, such as the hypersonic aircraft antenna systems, 5G/6G base stations, and others [13], [14].

**Figure 1:**
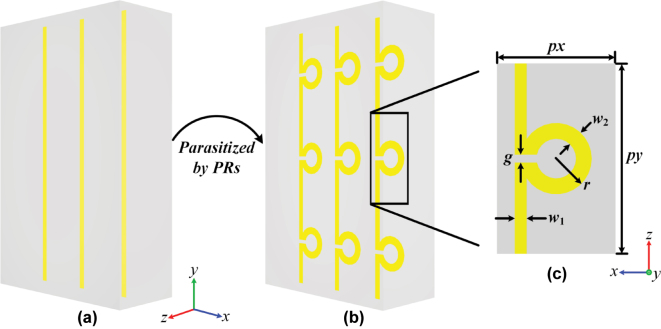
The LPR metasurface. (a) The LMS array and (b) the array parasitized by the PRs, which has been simply called the LPR metasurface here; (c) the top view of the unit of the LPR metasurface.

To solve these problems, we have proposed the LPR metasurface to open the broadband dual-band EM window at large angles in this manuscript. As shown in Figure 1(a) and (b), the LPR metasurface is designed by introducing “parasitic” rings (PRs) into the LMS array. In C band, due to the PRs’ characteristics, the LPR metasurface could conserve the LMSs’ plasma-like oscillation, and thus open a broadband window. In K band, where the plasma-like oscillation has almost disappeared, the PRs would generate a capacitive resonance. It could not only open an additional window, but also provides a great out-of-band suppression at the same time. In addition to the additional window, the PRs are structurally easy and integrated into the LMSs, which are different from the introduced resonators in related studies. It has conserved the LMSs’ simplicity and continuity, and thus could better meet processing and protective materials. The working mechanism has been demonstrated in detail through the spectrum comparison and the monitored field distribution, and then well verified by the extracted EM parameters. The performance has been verified through both measurement and simulation, where the measured results are basically consistent with the simulated ones.

## 2 Theoretical analysis

In this section, we have analyzed the condition of EM windows, where a dielectric plate is used to simulate the radome. To meet applications, we have selected CMCs as the plate’s matrix materials, *d* = 12.0 mm as its thickness, and C and K bands as the low-frequency (LF) and high-frequency (HF) working bands, respectively. Moreover, with the poor large-angle transparency much more serious at TE polarization, the study has focused on TE polarization here.

As expressed in (1) and (2), with the TE-polarized electrical field always along the *y*-axis for any incident angle, there are two conditions for the EM window at TE polarization. The details could be found in Reference [14]. Here, *ɛ*_1*i*_ and *μ*_1*i*_ are the relative permittivity and permeability of CMCs along the *i*-axis, respectively. For the empty CMC plate without any metasurface loaded, there are *ɛ*_1*x*_ = *ɛ*_1*y*_ = *ɛ*_1*z*_ = 3.1 and *μ*_1*x*_ = *μ*_1*y*_ = *μ*_1*z*_ = 1.0. Additionally, *ɛ*_0_ and *μ*_0_ represent the permittivity and permeability of air, respectively; *θ*_0_ represents the incident angle; *η*_0_ represents the eigen wave impedance of air; *φ* represents the waves’ propagation phase in the plate; *k*_0_ represents the wave number in the air; *f* represents the EM frequency.

**Figure 2:**
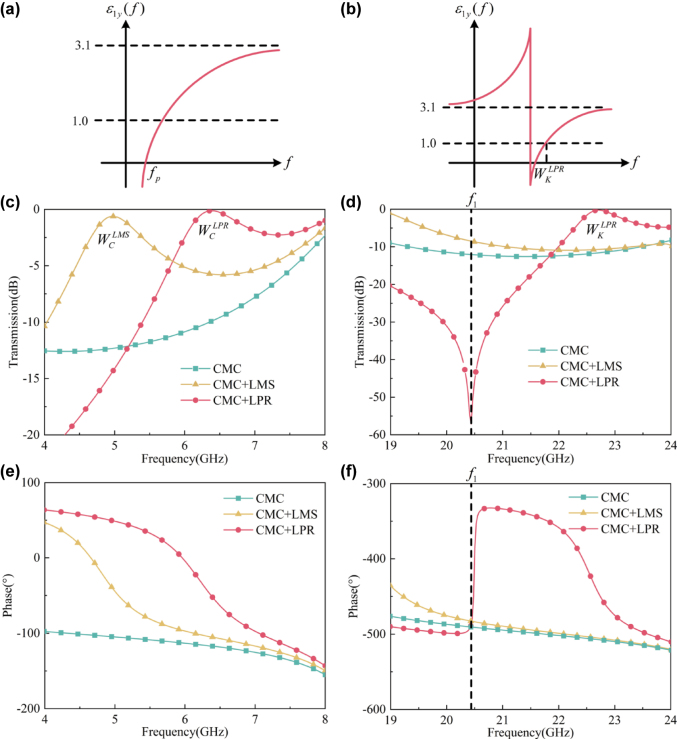
The function curves of *ɛ*_1*y*_(*f*) in (a) the plasma-like oscillation and (b) the capacitive resonance, respectively; the simulated TE-polarized transmission spectrum of the empty CMC plate, the plate loaded with the LMS array, and the one loaded with the LPR metasurface in (c) C and (d) K bands, respectively, with *θ*_0_ = 80°; the simulated TE-polarized transmission phase spectrum of the empty CMC plate, the plate loaded with the LMS array, and the one loaded with the LPR metasurface in (e) C and (f) K bands, respectively, with *θ*_0_ = 80°.

**Impedance match.** In addition to the expression, the nature of (1) and (2) should be known to guide the metasurface design. First, (1) represents the EM reflection coefficient on the CMC-air interface, which is zero, just the impedance match between CMCs and air [13], [14]. Obviously, if the loaded metasurface has no magnetic response (like the LMS array), there is (*μ*_1*x*_ = *μ*_1*y*_ = *μ*_1*z*_ = 1.0) and (1) is equivalent to *ɛ*_1*y*_ = 1.0. In such a condition, the transparent window is not only opened, but also angular stable, because (1) is independent of *θ*_0_. With the plasma-like oscillation to decrease the permittivity, the LMS array has been generally used for this method. In the plasma-like oscillation along the *y*-axis, *ɛ*_1*y*_ is dispersed as expressed in (3). Here *f* is the normalized frequency; *γ* is the EM loss; *f*_*p*_ is the plasma-like frequency, which is determined by the strips’ width and period [5]. The corresponding function curve of *ɛ*_1*y*_(*f*) could be drawn as Figure 1(a), where *ɛ*_1*y*_(*f*) = 1.0 at a frequency above *f*_*p*_. Additionally, just as shown in Figure 2(a), the plasma-like oscillation’s dispersion is gentler than that of other EM resonances (like electric and magnetic dipole resonances, and others), so the window through it could hold a wide bandwidth [28], [29], [30], [31]. So, we have designed the LMS array along the *y*-axis for the CMC plate, as shown in Figure 1(a), which is loaded in the plate’s middle layer for a wide transparent bandwidth.

However, just as shown in Figure 2(a) and discussed in introduction, the plasma-like oscillation has attenuated with the increasing frequency, so it could only work for the single-band window, but not the dual-band one. Additionally, in dual-band designs, it could only work for the LF window rather than the HF one. The reasons are as follows: First, the plasma-like oscillation would reduce the permittivity to be negative below *f*_*p*_, as shown in Figure 2(a). Therefore, if it is at HFs, the transmission at LFs would be greatly suppressed, and thus the LF window is broken [17], [18]. Second, the plasma-like oscillation’s EM response is weak as a non-resonant phenomenon, which is also why its dispersion is gentle. Therefore, with the substrate’s electrical thickness large at HFs, the LMSs in the middle layer might be too “far” away from the CMC-air interfaces, and thus could not regulate the waves (reflection coefficient) there. Thus, the impedance matching can’t be realized. Here, in C band, the plate’s relative electrical thickness is small (0.43*λ*_*C*_), and the above method is applicable. However, it’s large (1.56*λ*_*K*_) in K band, and the method isn’t applicable. *λ*_*C*_ and *λ*_*K*_ are the central wavelengths in C and K bands, respectively.

**Destructive interference.** (2) is the destructive interference of reflective waves, which is also the mechanism of AR films and half-wave walls [1], [2], [32], [33]. Obviously, it depends on the delay phase *φ*. So, it requires regulating the waves inside the plate, but not that on the interface, which is different from (1). Therefore, (2) is not subject to the plate’s electrical thickness and thus is applicable to the HF window. However, different from the impedance match, (2) well depends on *θ*_0_. So, the window through it is angular unstable, which is unsuitable for applications.

In summary, for the dual-band window, an EM mechanism other than the plasma-like oscillation should be introduced into the LMS to open the HF window. Moreover, for the angular stability, the best method is to achieve the impedance match at HFs. Thus, the introduced mechanism should also decrease the permittivity like the plasma-like oscillation, and be resonant for the substrate’s large electrical thickness at HFs.

(1) $$\frac{\eta_{0}}{\sqrt{\frac{\varepsilon_{1y}}{\mu_{1x}}-\sin^{2}\theta_{0}\frac{1}{\mu_{1y}\mu_{1x}}}}=\frac{\eta_{0}}{\cos\theta_{0}}$$

(2) $$\varphi =k_{0}\sqrt{\mu_{1x}\varepsilon_{1y}-\sin^{2}\theta_{0}\frac{\mu_{1x}}{\mu_{1z}}}d=m\pi ,\;m=0,1,2\ldots$$

(3) $$\varepsilon_{1y}\left(f\right)=3.1\left(1-\frac{f_{p}^{2}}{f^{2}+i\gamma f/2\pi }\right)$$

(4) $$\varepsilon_{1y}\left(f\right)=3.1\left(1.0-\frac{Af_{1}^{2}}{f^{2}-f_{1}^{2}+i\gamma f/2\pi }\right)$$

## 3 Metasurface design

**LPR metasurface.** Based on the above analysis, we have introduced split resonance rings (SRRs) into the LMS array. As shown in Figure 1(b), the SRR is integrated into the LMS, as if it has “parasitized” in the LMS. Therefore, we have called it the parasitic ring (PR) here, and the LMS array parasitized by PRs as the LPR metasurface. All the related structural parameters are shown in Figure 1(c), while their values are given in Table 1. Obviously, the PRs have conserved the LMS array’s simplicity and continuity, which are different from the introduced resonators, structurally complex and completely independent in related studies [17].

**Table 1:** The values of all the structural parameters.

*px*	*py*	*w*_1_	*w*_2_	*r*	*g*	*t*	*d*
3.0	4.8	0.3	0.4	0.9	0.2	0.02	12.0

**Figure 3:**
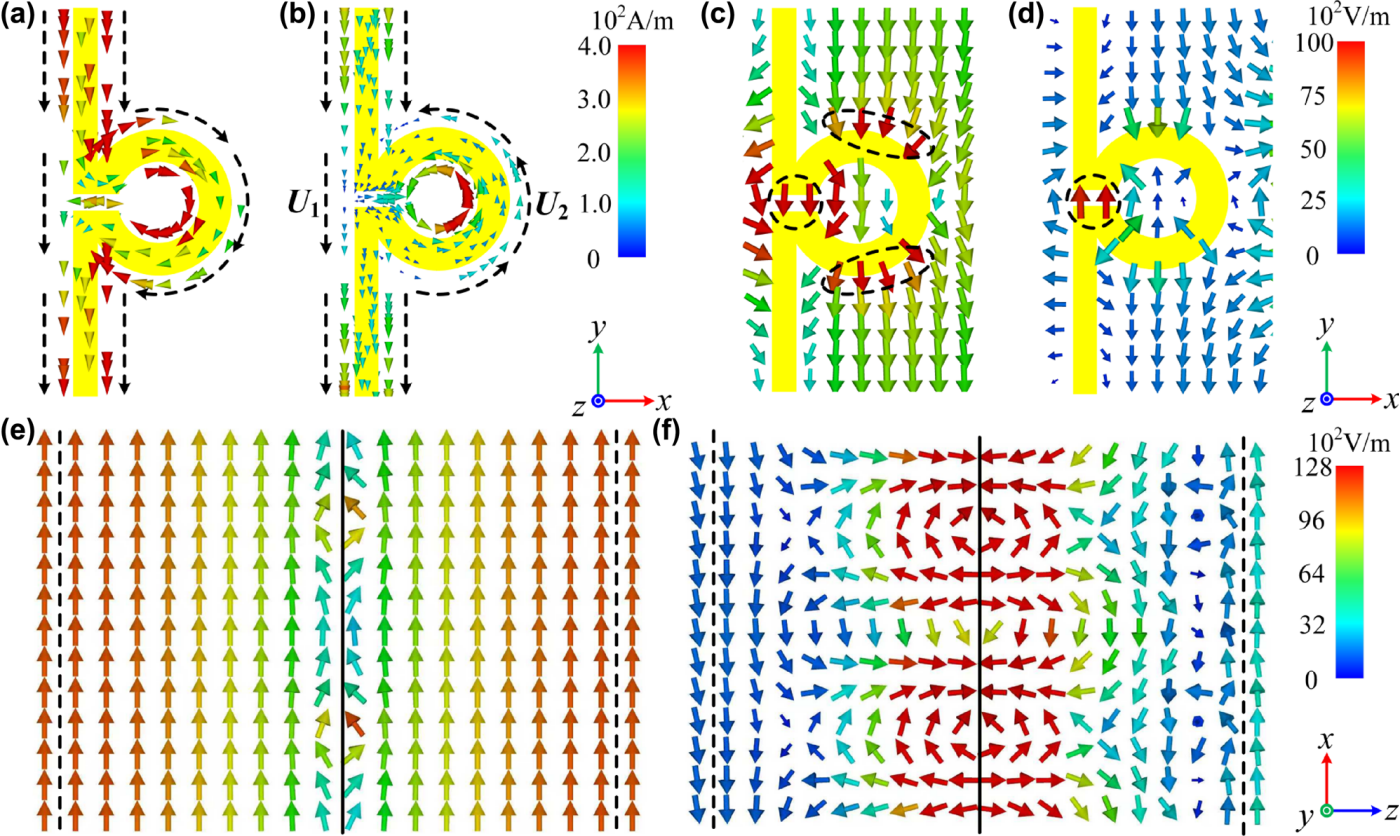
The monitored ((a) and (b)) surface currents, ((c) and (d)) electrical field on *xOy* plane, and ((e) and (f)) electric field on *xOz* plane at $W_{L}^{LPR}$ and *f*_1_, respectively, with TE polarization incident at *θ*_0_ = 80°.

**Working mechanism.** To demonstrate the design’s working mechanism, we have compared the simulated TE-polarized transmission and transmission phase spectrum of the empty CMC plate, the plate loaded with the LMS array, and the one loaded with the LPR metasurface with *θ*_0_ = 80° in C and K bands, respectively, as shown in Figure 2(c–f). Moreover, we have monitored the surface currents and electrical field in the LPR metasurface at the crucial frequencies as shown in Figure 3(a–f). In our simulation, the waves are incident from the *z*-axis’s positive direction.

In C band, just as discussed in theoretical analysis, the LMS array could generate the plasma-like oscillation, and thus open a broadband window at 5.0 GHz ( $W_{C}^{LMS}$ ) as shown in Figure 2(c). For the LPR metasurface, the LMSs’ continuity is conserved, although the PRs are introduced. Additionally, with the electrical size around 0.06 *λ*_*C*_, the PRs have no resonant response to the incident field. As shown in Figure 2(e), the transmission phase of the LPR-loading plate has no transition, just like that of LMS-loading and empty ones, which indicates none of the resonant response of the LPR metasurface. Therefore, the surface currents induced in LMSs could go through the PRs, and thus flow continuously, just as shown in Figure 3(a) (the black dotted arrows represent the currents’ direction). So, the LPR metasurface could preserve the LMSs’ plasma-like oscillation and the window through it. The window is denoted as $W_{C}^{LPR}$ (6.35 GHz) here, which corresponds to $W_{C}^{LMS}$ of the LMS array. As shown in Figure 3(e), the incident electrical field passes through both the upper and lower CMC-air interfaces with nearly no change at $W_{C}^{LPR}$ , which indicates the impedance match between CMCs and air. Here, the black dotted lines represent the interfaces, and the black solid line represents the LPR metasurface. For the frequency difference between $W_{C}^{LPR}$ and $W_{C}^{LMS}$ in Figure 2(c), it’s due to the additional induced electrical field around the PR as shown in Figure 2(g) (see the field circled by the black dotted line), which would influence the plate’s *ɛ*_1*y*_(*f*) in the plasma-like oscillation.

In K band, the plasma-like oscillation has almost disappeared. Therefore, there is no window in the LMS-loading plate’s spectrum as shown in Figure 2(d), and the induced currents in LMSs of the LPR metasurface are very weak as shown in Figure 3(b). However, as the wavelength shortened, the PR possesses an electrical size of around 0.25*λ*_*k*_, and thus generates a capacitive resonance. As shown in Figure 3(b), due to the gap being on only one side, the PR is asymmetric about the *y*-axis. Therefore, unequal electromotive forces (*U*_1_ and *U*_2_) would be excited on its two sides at TE polarization, thereby inducing a current of circulation. It can be seen that the induced circuit currents in PRs are in opposite directions of those in LMSs (see the black dotted arrows), which is due to the EM phase transition at the resonance. With the circulation current, the charges are accumulated in one side of the gap. At this time, the gap is just like a capacitor, and its two sides are the positive and negative electrodes, respectively. Thus, an electrical field is induced in the gap along the *y*-axis, as shown in Figure 3(d). Due to the induced electrical field, the plate’s *ɛ*_1*y*_ is dispersed as expressed in (3). Here, *A* represents the constant coefficient; *γ* represents the EM loss; *f* represents the normalized frequency;*f*_1_ represents the resonance frequency, which is determined by the total inductance and capacitance of the PR. The corresponding function curve has been drawn in Figure 2(b) with the loss ignored, where *ɛ*_1*y*_(*f*) has been increased below *f*_1_, reduced beyond *f*_1_, and reached infinity at it [27], [28], [29]. Meanwhile, as shown in Figure 3(f), due to the resonance’s strong response, the influence of the induced electrical field is throughout the whole plate and reaches the surface. Thus, it could realize the impedance match between the CMCs and air. So, there is *ɛ*_1*y*_(*f*) = 1.0 at a frequency beyond *f*_1_, where the impedance match is realized and the HF window is opened. Here, we have designed *f*_1_ at around 20.4 GHz by modifying the structural parameters in Table 1, where the detailed relationship between the parameters and spectrum is given in Supplement Materials. As shown in Figure 2(f), the transmission phase of the LPR-loading plate has a transition around 180° at 20.4 GHz, which indicates a resonance there. In such a condition, the HF window ( $W_{K}^{LPR}$ ) is at 22.3 GHz as shown in Figure 2(d). Moreover, the strong vibration at *f*_1_ has led to a transmission zero there, which is between the LF and HF windows and of great significance to applications.

**Figure 4:**
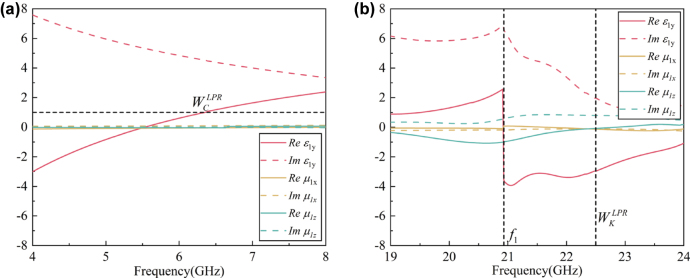
The extracted real and imaginary parts of the LPR-loading plate’s *ɛ*_1*y*_, *μ*__1*x*__, and *μ*__1*z*__ in (a) C and (b) K bands, respectively.

As shown in Figure 4(a) and (b), in order to verify the above working mechanism, we have extracted the related EM parameters (*ɛ*_1*y*_, *μ*_1*x*_, and *μ*_1*z*_) of the LPR-loading plate in C and K bands, respectively, through the method in Reference [34]. In C band, as shown in Figure 3(a), the *ɛ*_1*y*_ of the LPR-loading plate shows a dispersion similar to that of the plasma-like oscillation (see Figure 2(a)), while *μ*_1*x*_ and *μ*_1*z*_ are basically constant. Moreover, there is *ɛ*_1*y*_ = 1.0 at around $W_{C}^{LPR}$ . It has been proven that $W_{C}^{LPR}$ is through the plasma-like oscillation of the LPR metasurface. In K band, the CMC plate has a large electrical thickness as discussed before, so the LPR-loading plate couldn’t be regarded as a homogeneous medium. Thus, the method in Reference [34] couldn’t work for it. Therefore, in K band, we have only extracted the parameters of the LPR-loading plate’s middle layer, which is chosen to be 3.0 mm-thick according to *λ*_*K*_. As shown in Figure 4(b), the layer’s *ɛ*_1*y*_ is in a dispersion similar to that in Figure 2(b) with *f*_1_ ≈ 20.9 GHz, and well reduced near $W_{K}^{LPR}$ . The frequency difference between *f*_1_ in Figure 4(b) and that in Figure 2(f) is due to the different thicknesses of the plate, which would influence the resonance frequency in the spectrum. Additionally, *μ*_1*z*_ is weakly dispersed and reduced near *f*_1_, while *μ*_1*y*_ is nearly constant. Here, the dispersion of *μ*_1*z*_ is due to the magnetic field induced by the circulation current in the PR’s center. Considering the whole plate, although the extracted result of the middle layer here could not correspond to the capacitive resonance well, it could still prove $W_{K}^{LPR}$ is through the capacitive resonance.

## 4 Performance

**Figure 5:**
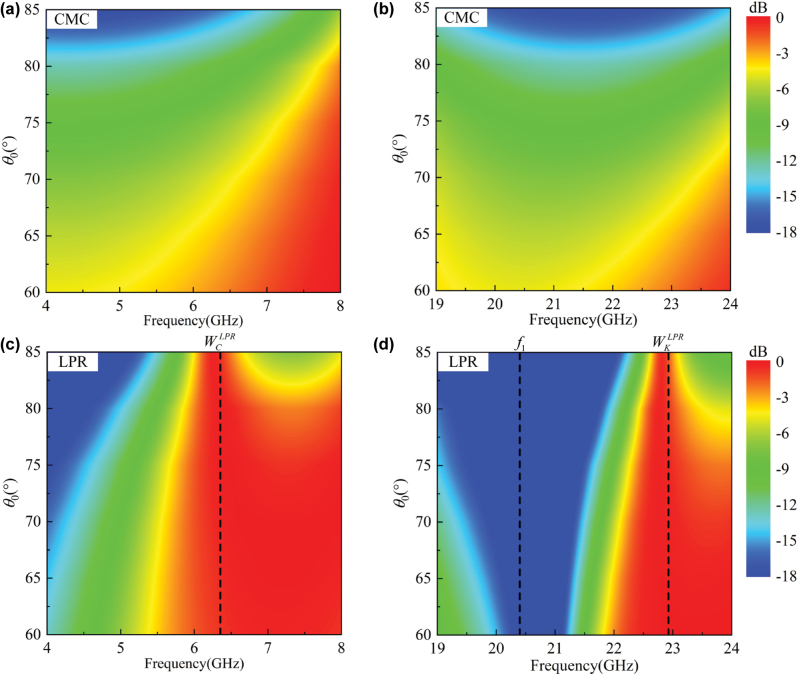
The simulated TE-polarized transmission spectrum of the empty CMC plate in (a) C and (b) K bands, respectively, within *θ*_0_ ∈ [60°–85°]; the simulated TE-polarized transmission spectrum of the plate loaded with the LPR metasurface in (c) C and (d) K bands, respectively, within *θ*_0_ ∈ [60°–85°].

Due to the angular stability of the impedance match, $W_{C}^{LPR}$ and $W_{K}^{LPR}$ could be effective within a wide large-angle area (*θ*_0_ *ɛ* [60°–85°]). In order to show the LPR metasurface’s performance of the dual-band EM window at large angles, we have compared the simulated TE-polarized transmission spectrum of the empty CMC plate and the LPR-loading one in C and K bands, within *θ*_0_ *ɛ* [60°–85°], respectively. As shown in Figure 5(a) and (c), after loading the LPR metasurface, an obvious transparent window is opened on the CMC plate at about 6.35 GHz ( $W_{C}^{LPR}$ ), where the maximum transmission is 99.8 %. Similarly, as shown in Figure 4(b) and (d), there is also a window opened in K band, which is at around 22.3 GHz ( $W_{K}^{LPR}$ ), and the maximum transmission is 93.9 %. In addition to the large EM transmission, $W_{C}^{LPR}$ and $W_{K}^{LPR}$ both have a great angular stability in the spectrum due to the impedance match, which is consistent with theoretical analysis. Moreover, due to the capacitive resonance, a transmission zero has been introduced at around 20.9 GHz (*f*_1_), which has provided a great out-of-band suppression between the two working bands. By the way, it is obvious that the maximum transmission at $W_{K}^{LPR}$ is lower than that at $W_{C}^{LPR}$ . It is because the metal has large ohmic loss at around the resonance, which is caused by strong induced currents.

**Comparison.** To further demonstrate the great performance of the LPR metasurface, we have compared it with other related studies of transparent metasurfaces. Here, the effective bands (EBs) and fractional bandwidths (FBWs) at *θ*_0_ = 80° of all the designs are given in Table 2, where the effective transmission is still defined as > −3 dB. The FBW is expressed as (*f*_*h*_ − *f*_*l*_)/*f*_*c*_, where *f*_*l*_ *,* *f*_*h*_, and *f*_*c*_ represent the lower, higher, and central frequencies of the effective transparent band, respectively. Obviously, the LPR metasurface’s LF- and HF-FBWs have been calculated as (5.95–7.32 GHz) 20.6 % and (22.55–23.35 GHz) 3.7 %, respectively, which are both wider than those in other studies. Meanwhile, the LF-FBW is much wider than the HF-one. As discussed in theoretical analysis, this is due to the plasma-like oscillation’s dispersion being gentler than that of the capacitive resonance, as shown in Figure 2(a) and (b) [5]. Additionally, it can be seen that the dispersion of *ɛ*_1*y*_(*f*) becomes more intense as the frequency gets closer to *f*_1_ in Figure 2(b), which further reduces the bandwidth of $W_{K}^{LPR}$ .

**Table 2:** OBs and FBWs of the LPR metasurface and other related works at *θ*_0_ = 80°.

	OB	FBW
LPR	5.95–7.32 GHz	20.6 %
	22.55–23.35 GHz	3.7 %
Reference [18]	5.68–6.43 GHz	12.4 %
Reference [11]	33.01–35.29 GHz	6.7 %
Reference [9]	277.12–292.42 GHz	5.4 %
Reference [12]	76.31–78.94 GHz	3.4 %
Reference [35]	6.85–7.03 GHz	2.6 %

**TM-polarized performance.** To be more applicable, the metasurface should work for both TE and TM polarization. Therefore, the LPR metasurface’s TM-polarized performance is discussed and improved. The improvement method is introducing a metagrating along the *x*-axis, where the details are given in Supplement Materials.

**Figure 6:**
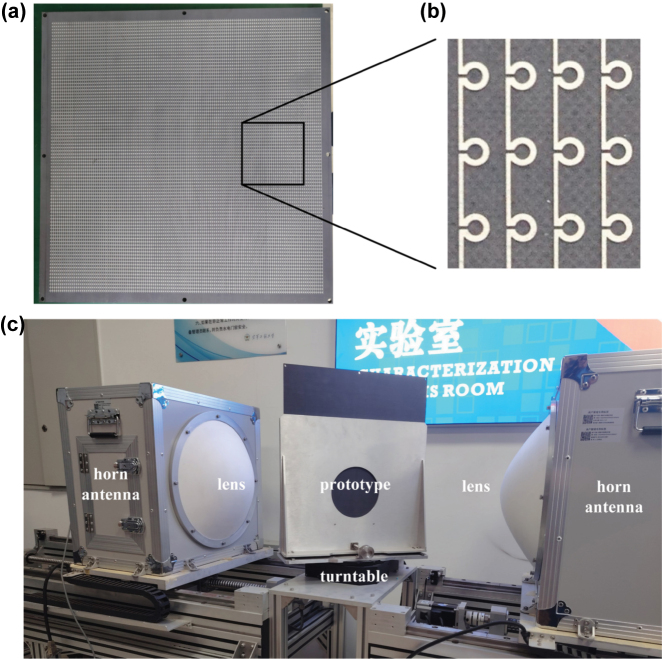
The measurement. (a) The processed CMC plate with the LPR metasurface etched on one of its surfaces and (b) the locally enlarged image of its surface; (c) the measured environment.

**Measurement.** To further verify the performance of the LPR metasurface, the prototypes were designed, processed, and measured. First, we have processed a 6.0 mm-thick CMC plate, and etched the LPR metasurface on one side of it through the printed circuit board technology. Then, we have stuck the above plate with the other empty plate of the same thickness through the glass cement, with the metasurface in the sandwich layer. The processed prototype and the local enlarged image of its sandwich layer are shown in Figure 6(a) and (b), respectively. In addition, a 12.0 mm-thick empty CMC plate is processed as a comparison prototype. To match the measured equipment, all prototypes are processed as 400 mm × 400 mm.

**Figure 7:**
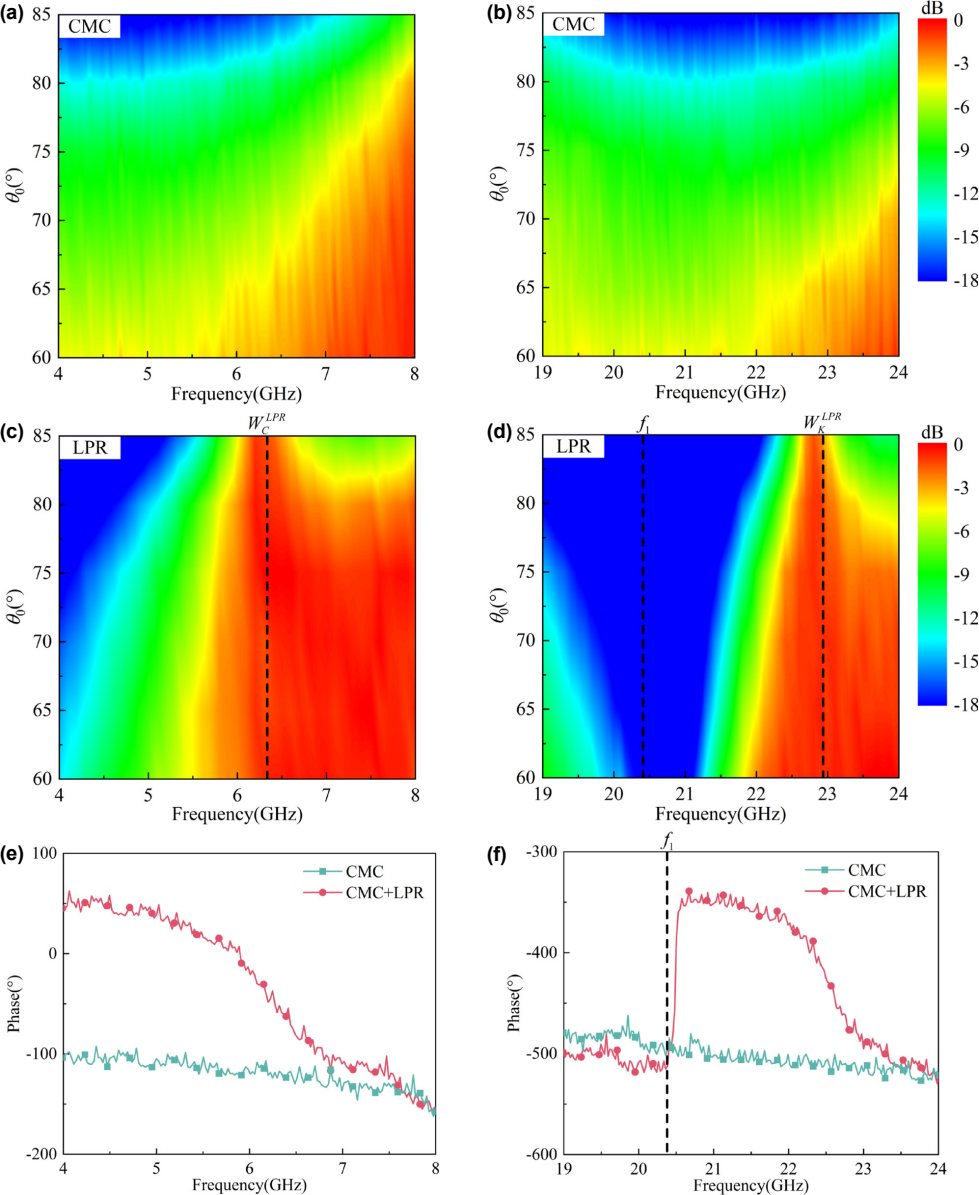
The measured TE-polarized transmission spectrum of the empty CMC plate in (a) C and (b) K bands, respectively, within *θ*_0_ ∈ [60°–85°]; the measured TE-polarized transmission spectrum of the LPR-loading plate in (a) C and (b) K bands, respectively, within *θ*_0_ ∈ [60°–85°]; the measured TE-polarized transmission phases of the empty CMC plate and the LPR-loading plate in (e) C and (f) K bands, respectively, at *θ*_0_ = 80°.

As shown in Figure 6(c), the two prototypes were measured in the lens-antenna measurement system, respectively. Here, the horn antennas were used as the transmitting and receiving ports, and the prototype was positioned on the turntable between the two lenses. The measured results of the two prototypes are shown in Figure 7(a–d), which are basically consistent with the simulation ones. Meanwhile, the transmission phases of them are also measured at *θ*_0_ = 80°, which are also in accord with the simulated ones in Figure 2(e) and (f). By the way, the error between the measured and simulated results may be due to the clutter and losses in transmission lines.

## 5 Conclusions

In this manuscript, we have proposed to introduce PRs into the LMS array to open the dual-broadband EM window at large incident angles, where the final design is called the LPR metasurface. First, we have carried out the theoretical analysis and derived the two conditions to open the TE-polarized window on a dielectric plate. The nature of the two conditions is further analyzed to guide the metasurface design, which involves the impedance matching and destructive interference, respectively. Based on the analysis, we have proposed to introduce PRs into the LMS array, thus designing the LPR metasurface. In C band, the LPR metasurface could conserve the LMSs’ plasma-like oscillation, and thus open a broadband window through the impedance matching. In K band, although the plasma-like oscillation has almost disappeared, the PRs could generate a capacitive resonance, which could open an additional window also through the impedance match. Through the transmission spectrum and the monitored field distribution, the working mechanism has been demonstrated in detail. Moreover, to verify it, we have extracted the LPR-loading plate’s EM parameters, which are basically consistent with the demonstrated mechanism. The LPR’s performance has been verified through both the simulation and measurement, where the measured and simulated results are in agreement with each other.

Different from the introduced resonators in related studies, the PRs have conserved the LMSs’ simplicity and continuity in the LPR metasurface, and thus could better meet processing and protective materials in EM windows. In addition, with the great performance at large angles, the LPR metasurface may find wide applications in hypersonic aircraft radars, 5G/6G base stations, and others.

## 6 Methods

**Simulation Section.** With the assistance of the commercial software CST microwave studio, the S_Zmax(1),Zmin(1)_ (transmission) of unit cells of proposed anti-reflective metasurface and the substrate is simulated with frequency domain solver and boundary conditions that X-unit cell, Y-unit cell and Z-open add space. And the surface current and energy flow distributions are obtained by the field monitor.

## Supplementary Material

Supplementary Material Details
