# Supplementary material for: Long-metallic-strip array with parasitic rings: an efficient metasurface for dual-broadband electromagnetic window at large angles
Source: Nanophotonics. 2025 Oct 16;14(22):3579–89. doi: 10.1515/nanoph-2025-0157 (PMC12592635; doi:10.1515/nanoph-2025-0157)
Supplement: Supplementary file 1 — Supplementary Material Details [file j_nanoph-2025-0157_suppl_001.docx]

Supplement Materials

1. **Structural Parameters Design**

Just as discussed in the text, $W_{C}^{LPR}$ and $W_{K}^{LPR}$ are due to the plasma-like oscillation and the capacitive resonance, respectively. Thus, the ideal frequencies of $W_{C}^{LPR}$ and $W_{K}^{LPR}$ are equivalent to the ideal $f_{p}$ and $f_{1}$. Meanwhile, the relationship between $f_{1}$ ($f_{p}$) and structural parameters is clear, according to the mechanisms of the plasma-like oscillation and the capacitive resonance. Based on the relationship, the structural parameters could be easily obtained. In this section, to demonstrate the relationship, the LF- and HF-TE-polarized transmission spectrum of the LPR-loading plate with different $r$, $g$, $w_{2}$, and $w_{1}$ is given in Fig. S1(a-h), respectively, with $\theta_{0}$= 80$^{\circ}$.

As shown in Fig. S1(a), $W_{C}^{LPR}$ moves to lower frequencies as $r$ increases. It is because the increase of $r$ hinders the flow of currents in LMSs at LFs, and thus decreases $f_{p}$ (the phenomenon is equivalent to the decrease of $w_{1}$). Meanwhile, as shown in Fig. S1(b), $W_{K}^{LPR}$ also moves to lower frequencies with the increase of $r$. It is due to the increase in $r$, which increases the PR’s inductance, thereby decreasing $f_{1}$.

As shown in Fig. S1(c), $W_{C}^{LPR}$ doesn’t change with the alteration of $g$. It is because the change of $g$ has almost no influence on the flow of currents in LMSs at LFs, and thus has no influence on $f_{p}$. Meanwhile, as shown in Fig. S1(d), $W_{K}^{LPR}$ also moves to lower frequencies as $g$ decreases. It is because the decrease in $g$ increases the PR’s capacitance, thereby decreasing $f_{1}$.

As shown in Fig. S1(e), $W_{C}^{LPR}$ moves to lower frequencies as $w_{2}$ decreases. It is because the decrease of $w_{2}$ is equivalent to the decrease of $w_{1}$ with the connection of LMSs and PRs, which decreases $f_{p}$. Meanwhile, as shown in Fig. S1(f), $W_{K}^{LPR}$ also moves to lower frequencies with the decrease of $w_{2}$. It is due to the decrease of $w_{2}$, which increases the PR’s inductance, thereby decreasing $f_{1}$.

As shown in Fig. S1(e), $W_{C}^{LPR}$ moves to lower frequencies as $w_{1}$ decreases. It is because the decrease of $w_{1}$ is also equivalent to the decrease of $w_{2}$ with the connection of LMSs and PRs, which decreases $f_{p}$. Meanwhile, as shown in Fig. S1(f), $W_{K}^{LPR}$ also moves to lower frequencies with the decrease of $w_{1}$. For the same reason, it is because the decrease of $w_{1}$ increases the PR’s inductance (equivalent to the decrease of $w_{2}$), thus decreasing $f_{1}$.

In summary, the ideal structural parameters could be obtained based on the above relationship. Meanwhile, the relationship is consistent with the LPR metasurface’s working mechanism.


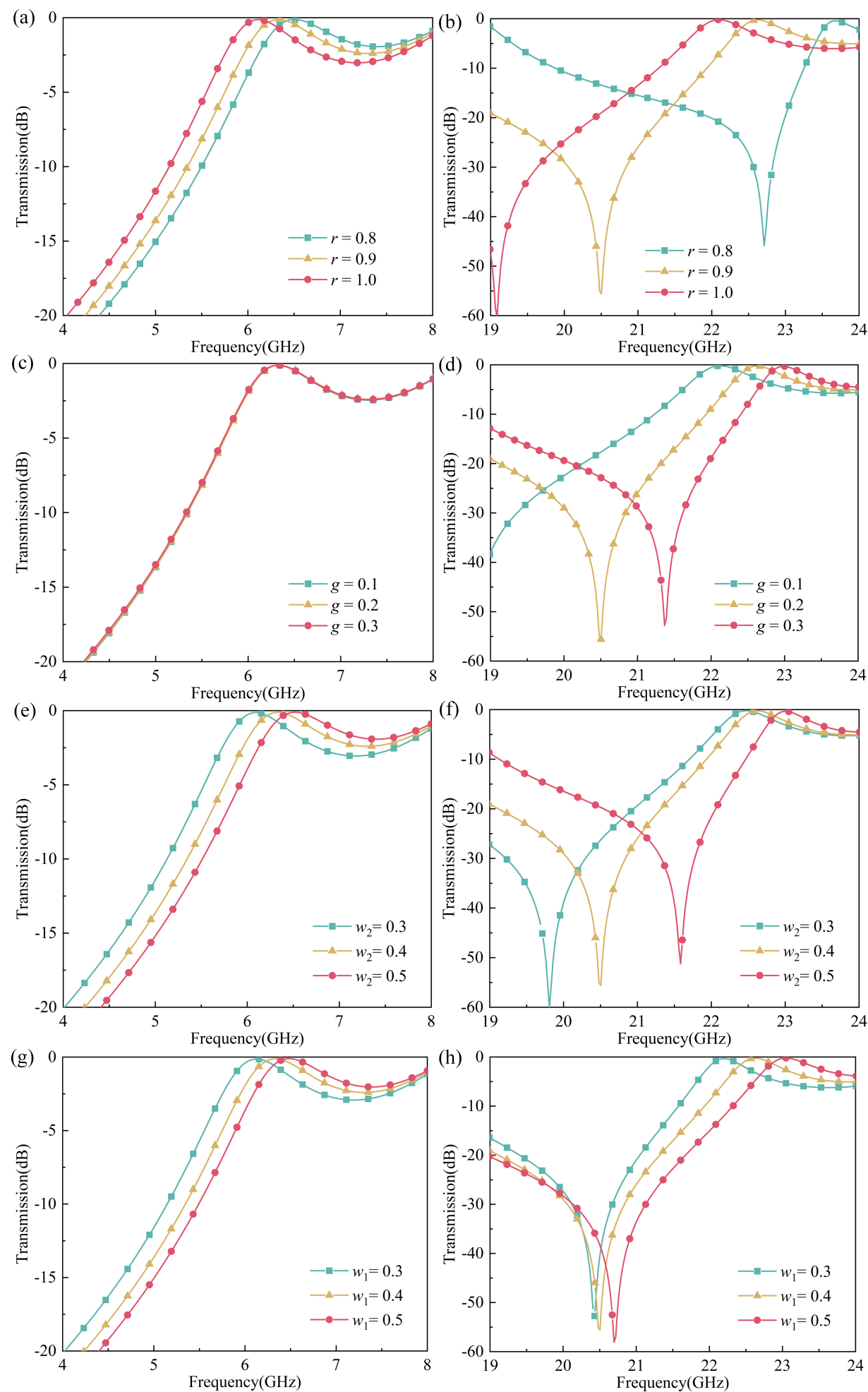


**Fig. S1.** The LF- and HF-TE-polarized transmission spectrum of the LPR-loading plate with different ((a) and (b)) $r$, ((c) and (d)) $g$, ((e) and (f)) $w_{2}$, and (((g) and (h))) $w_{1}$ respectively, with $\theta_{0}$= 80$^{\circ}$.


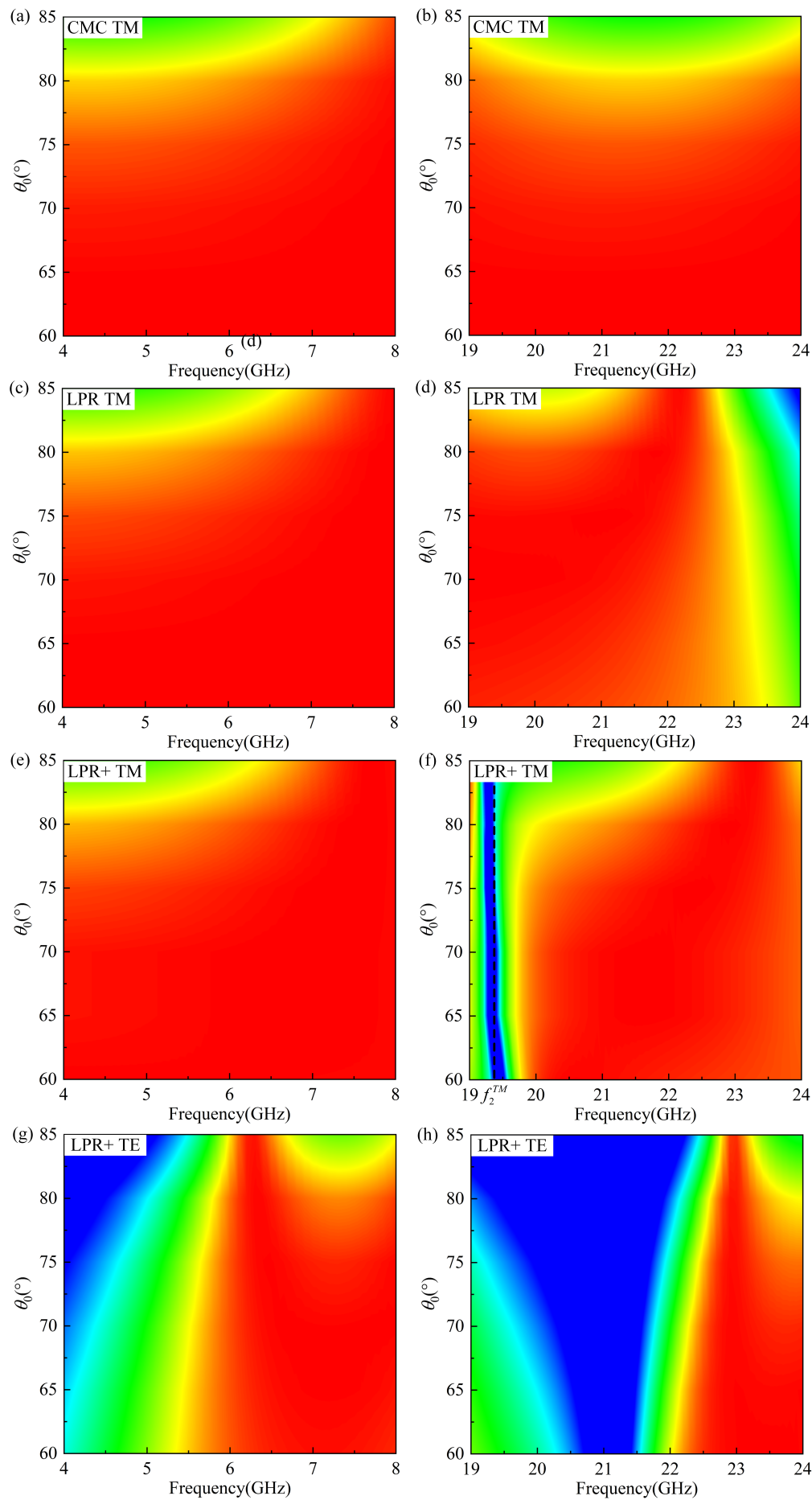


**Fig. S2.** The TM-polarized transmission spectrum of the empty CMC plate in (a) C and (b) K bands, respectively; The TM-polarized transmission spectrum of the LPR-loading plate in (c) C and (d) K bands, respectively; The TM-polarized transmission spectrum of the LPR$+$-loading plate in (e) C and (f) K bands, respectively; The TE-polarized transmission spectrum of the LPR$+$-loading plate in (g) C and (h) K bands, respectively.

**2. TM-polarized Performance**

Here, the LPR metasurface’s TM-polarized performance and the optimization method for it are discussed, where the TM-polarized magnetic field is defined as along the *y-*axis for any incident angle (the electrical field is in the *xOz* plane). First, for the bare CMC substrate, with the Brewster angle, the TM-polarized transmission is generally higher than the TE-polarized one at large angles, as shown in Fig. S2(a) and (b).

After loading the LPR metasurface, the plate could still have high large-angle TM-polarized transmission within the effective working band of $W_{C}^{LPR}$, as shown in Figure. S2(c). It’s due to the LMSs and PRs both having nearly no EM response for TM-polarized incidence at LFs. However, as shown in Fig. S2(d), the plate’s high transmission is influenced at $W_{K}^{LPR}$. It is due to the PRs having a capacitive resonance along the *x*-axis at TM-polarized, where the resonance frequency is around 27.8GHz (outside the observed frequencies). To solve the problem, a metagrating is introduced into the LPR metasurface as shown in Fig. S3(a), where the newly designed metasurface is called the LPR$+$ metasurface here. As shown in Fig. S3(b), the metagrating is composed of short metallic strips along the *x*-axis, which are loaded between the two adjacent PRs. Due to the small electrical size, the short strips have no EM response at LFs. Thus, as shown in Fig. S2(e), the introduced metagrating not only doesn’t break the plate’s high TM-polarized transmission at $W_{C}^{LPR}$, but actually improves it slightly. Meanwhile, in K band, the strip has a dipole resonance along the *x*-axis at $f_{2}^{TM}\approx$19.3GHz as shown in Fig. S2(f). Importantly, due to the dipole resonance, the plate’s transmission is well improved at $W_{K}^{LPR}$, which was originally broken by the PRs’ capacitive resonance. Additionally, the metagrating has nearly no influence on the LPR metasurface’s TE-polarized performance, because the short strips have nearly no EM response at TE polarization. As shown in Fig. S2(g) and (h), the TE-polarized transmission spectrum of the LPR$+$-loading plate is basically consistent with that of the LPR-loading one.

In summary, the designed LPR$+$ metasurface could work well for both TE and TM polarization through the above optimization, which has a great application value.


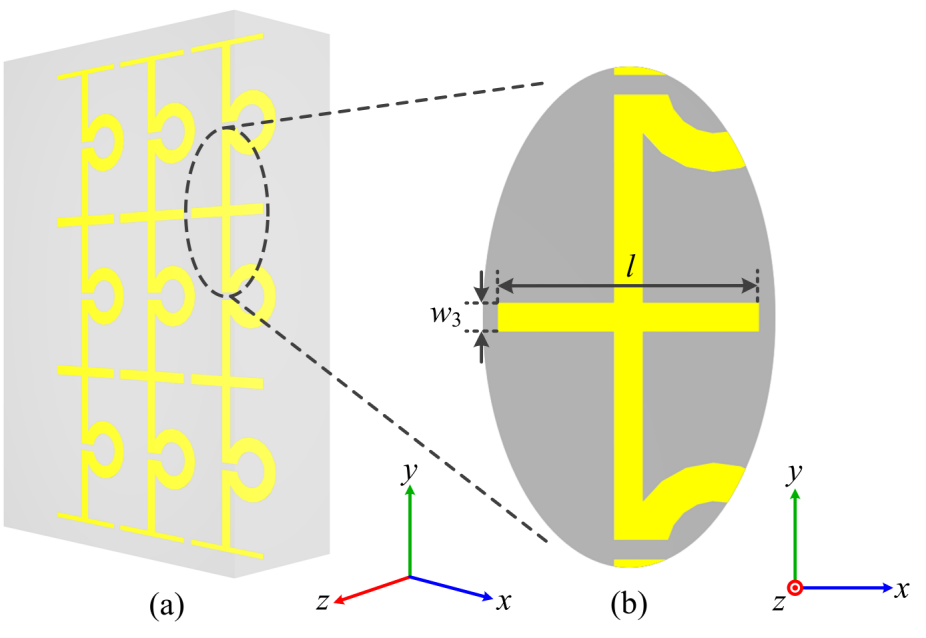


**Fig. S3.** (a) The LPR metasurface introduced with the metagrating, which is called the LPR$+$ metasurface here; (c) The top view of the unit of the LPR$+$ metasurface, where $l$ = 2.7mm is the strips’ length, and $w_{3}$ = 0.3mm is the strips’ width.
